# The association of high sensitivity C-reactive protein and incident Alzheimer disease in patients 60 years and older: The HUNT study, Norway

**DOI:** 10.1186/s12979-017-0106-3

**Published:** 2018-01-22

**Authors:** Jessica Mira Gabin, Ingvild Saltvedt, Kristian Tambs, Jostein Holmen

**Affiliations:** 1 HUNT Research Centre, Department of Public Health and Nursing, Norwegian University of Science and Technology (NTNU), Forskningsveien 2, 7600 Levanger, Norway; 2 Department of Neuromedicine and Movement science, NTNU, the Faculty of Medicine and Health, Post Office Box 8905, 7491 Trondheim, Norway; 3 Department of Geriatrics, St. Olav University Hospital, Post Office Box 3250, 7006 Trondheim, Norway; 4 Division of Mental Health, Norwegian Institute of Public Health, Post Office Box 4404, Nydalen, 0403 Oslo, Norway

**Keywords:** Epidemiology, Low-grade inflammation, High sensitivity C-reactive protein, Alzheimer disease

## Abstract

**Background:**

With ageing, long-standing inflammation can be destructive, contributing to development of several disorders, among these Alzheimer’s disease (AD). C-reactive protein (CRP) is a relatively stable peripheral inflammatory marker, but in previous studies the association between highly sensitive CRP (hsCRP) and AD have shown inconsistent results. This study examines the association between AD and hsCRP in blood samples taken up to 15 years prior to the diagnoses of 52 persons with AD amongst a total of 2150 persons ≥60 years of age.

**Results:**

Data from Norway’s Nord-Trøndelag Health Study (HUNT 2) and the Health and Memory Study (HMS) were linked. The participants had an average age of 73 years, and diagnosed with AD up to 15 years [mean 8.0 (±3.9)] following hsCRP measurement. Logistic regression models showed an adverse association between hsCRP and AD in participants aged 60-70.5 (odds ratio: 2.37, 95% CI: 1.01-5.58). Conversely, in participants aged 70.6-94, there was an inverse association between hsCRP and AD (odds ratio: 0.39, 95% CI: 0.19-0.84). When applying multivariate models the findings were significant in individuals diagnosed 0.4-7 years after the hsCRP was measured; and attenuated when AD was diagnosed more than seven years following hsCRP measurement.

**Conclusions:**

Our study is in line with previous studies indicating a shift in the association between hsCRP and AD by age: in adults (60-70.5 years) there is an adverse association, while in seniors (>70.6 years) there is an inverse association. If our findings can be replicated, a focus on why a more active peripheral immune response may have a protective role in individuals ≥70 years should be further examined.

## Background

Pre-clinical and clinical studies have shown that the immune system contributes and drives Alzheimer disease (AD) pathogenesis [1]. Inflammatory proteins found outside of the brain have also been shown to be elevated in patients with AD [2]. With ageing, long-standing inflammation can be destructive, contributing to development of several disorders [3, 4].

A minor elevation in inflammatory markers in blood is termed low-grade inflammation, where the body is constantly under very mild chronic elevation, but not to the extent of acute inflammation [5]. Low grade inflammation is recognized as an important contributor to the pathophysiology of hypertension, to the initiation and progression of atherosclerosis and the development of cardiovascular disease (CVD) [6]. C-reactive protein (CRP) is a relatively stable peripheral inflammatory marker that has been used as a marker of low-grade inflammation, and the highly sensitive assay (hsCRP) has been shown to be moderately elevated in acute myocardial infarction, coronary artery disease, metabolic syndrome, neurodegenerative diseases, and hypertension [7–10]. Since several CVD have been shown to share risk factors for developing dementia, a number of studies have examined whether there is an association between low-grade peripheral inflammation and AD [11, 12]. However, previous epidemiological studies examining hsCRP and AD revealed conflicting findings. Studies examining hsCRP during midlife showed adverse associations, where moderate elevations of hsCRP were increased in persons who developed AD later in life [13]. In contrast, studies examining older participants published that higher plasma levels of hsCRP was associated with a lower risk for AD and all-cause dementia, and authors questioned whether this could be attributed to a genetic phenotype for successful aging [14, 15]. Some studies examining gene expression have shown down-regulation of immune response genes in brain regions of cognitively impaired oldest-old persons and up-regulation in cognitively intact individuals of same age [16, 17]. Locascio et al. found that low levels of hsCRP were associated with more rapid progression of illness, whereas Nilsson et al. found that although CRP was overall lower in persons with AD, elevated CRP was associated with shorter survival time [18, 19]. Other studies show the opposite, that high hsCRP levels were associated with cognitive decline [2, 13, 20, 21]. However, previous studies were based on relatively small samples and short observation time.

In Nord-Trøndelag County, Norway, a large population based health study (the HUNT Study) combined with a registry of patients with dementia, provide data suitable for long follow-up time. The aim of this study was therefore to examine the association between hsCRP and AD in blood samples taken up to 15 years prior to the AD diagnosis amongst HUNT Study participants in Nord Trøndelag County over the age of 60 years.

## Methods

### Study population and data collection

The HUNT Study is a voluntary health survey offered to all residents in Nord-Trøndelag County (N~130,000). The region is approximately the size of Wales, rural, and located in central Norway. The HUNT Study consists of three population-based cohorts examining in total 125,000 residents during the span of three decades; HUNT 1 (1984-1986); HUNT 2 (1995-1997) and HUNT 3 (2006-2008). The HUNT Study has examined a large number of public health issues, like somatic and mental illnesses, quality of life, social factors, life style and other health determinants. The general methods for data collection were similar in all three HUNT surveys: several questionnaires, clinical measurements and collection of blood and urine samples. Participant’s age was obtained from the national population registry. History of myocardial infarction (MI), stroke, angina, diabetes mellitus (DM), smoking, and subjective health status were self-reported by participants. Clinical measurements were conducted in survey stations following standardized protocols. Pulse, systolic and diastolic blood pressure were measured three times using a Dinamap 845XT (Critikon) based on oscillometry. Body mass index (BMI) was based on height and weight measured with the participants wearing light clothes without shoes: height to the nearest centimeter and weight to the nearest half kilogram. Blood samples measuring non-fasting glucose, creatinine, triglycerides, and cholesterol used in the present study were collected at the health survey stations and transported to the biobank in well-described methods, that are published in detail previously [22, 23].

### hsCRP measurement

**Fig. 1:**
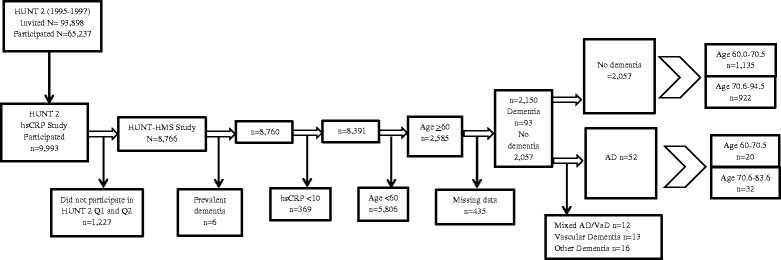
Flow-chart of the HUNT-HMS study sample examining high sensitivity C-reactive protein (hsCRP)

During HUNT 2 (*n* = 65,237) hsCRP measurement was measured in a subsample. For practical reasons, participants from four neighboring municipalities around the biochemical laboratory assaying hsCRP were selected randomly, and 9993 had their hsCRP measured (Fig. 1). The present study selected participants who had their hsCRP measured, returned the two main HUNT 2 questionnaires (*n* = 8766), and did not have prevalent dementia at time of survey participation (*n* = 8760). As hsCR*P* values can rise during active systemic infections or in acute inflammatory processes, we included only participants with hsCRP values less than 10 (*n* = 8391). Finally, we included only participants aged 60 and over (*n* = 2585) who had complete covariate data, which resulted in 2150 individuals who encompass the study sample. Non-fasting serum was stored at negative 80 degrees Celsius and measured two years after serum collection. The analysis were performed at a biomedical laboratory using the CRP (Latex) US (Hoffman-La Roche AG, Switzerland) standard assay for CRP analysis. Assay reproducibility was tested by the assay provider (Hitachi/Roche) and has run within [% coefficient of variation (CV) 0.43-1.34] and between days (%CV 2.51-5.70), in addition to running a method comparison (*r* = 0.996) [24].

### Dementia ascertainment

The Health and Memory Study of Nord-Trøndelag (the HMS Study) collected retrospectively data on individuals with dementia from the two regional hospitals between 1995 and 2010. Additionally, residents in all nursing homes in the region were examined for dementia between 2010 and 2011and ascertained by clinicians. The data collection has been more extensively described previously [25]. Briefly, two panels encompass the HMS study: a hospital and a nursing home panel. Ascertainment was uniform amongst panels and evaluated by clinicians confirming ICD-10 diagnostic criteria for AD, vascular dementia (VaD), and a mixture of these (mixed AD/VaD) based on clinical examination, patient and caregiver history and diagnostic imaging. Time of diagnosis was determined at assessment by clinicians and, if unknown, based on the initial documented examination date. The eleven-digit personal identification number given to each Norwegian resident linked the participants in HUNT 2 and individuals diagnosed with dementia in the HMS Study. Ninety-three HMS participants had hsCRP values less than ten and complete covariate data, of which 52 were diagnosed with AD; and are the focus of the present study. An additional 13 individuals were diagnosed with VaD, 12 mixed AD/VaD, and 16 with dementia of other causes.

### Data analysis

Participant’s age was used as a continuous variable in analyses. Supplemental analyses were used to examine a significant interaction effect and created by dichotomizing participants >60 in equal groups <70.6 and ≥70.6. Level of education was categorized according to primary (seven years or less), secondary (seven to nine years), and upper secondary education (>ten years). The average of the second and third blood pressure measurement was used in analyses. Non-fasting glucose, cholesterol, triglycerides and creatinine were scored as continuous variables. The independent-samples t-test and Pearson’s chi squared were used to compare the means between groups for continuous and categorical variables, and Mann-Whitney (MW) for comparisons between cases and non-cases examining hsCRP levels and potential covariates. HsCRP was examined in analyses as a continuous variable. The values of hsCRP were positively skewed and log transformations were used in all analyses and were less skewed; but neither distributions were normal. We used binary logistic models to estimate odds ratios (OR) with 95% confidence intervals (CI) for the associations of hsCRP to the incidence of AD, all-cause dementia, and non-AD dementia. Four sets of logistic regression models were performed for each endpoint in a hierarchy. Effect modification was examined by testing the statistical significance for age x hsCRP and sex x hsCRP in multivariable models. Additional analyses were performed to examine whether time to ascertainment influenced the association by splitting the sample equally in two according to the number of years to diagnosis from baseline. All statistical analyses were performed using SPSS, version 24.

## Results

The participants in the present study had an average age of 73 years and were diagnosed with AD up to 15 years [mean 8.0 (±3.9)] following hsCRP measurement. The characteristics of the study sample are shown in Table 1. Mean hsCRP are shown in their original values. Levels of hsCRP were significantly lower in the AD group ≥70.6 than in the reference group. Except from the age and sex differences, there were no significant differences between the study groups regarding other biomarkers, education, and history of MI, angina, stroke or DM.

**Table 1:** Characteristics of participants with no dementia (*n* = 2057) and participants with Alzheimer disease (*n* = 52) at the time of HUNT2 (baseline)

HUNT 2 (1995-1997)	No dementia	Alzheimer disease	*P* value^a^
Total study population, n	2057	52	
Sex, Female, n (%)	1120 (54.4)	34 (65.4)	.02
Age at HUNT 2 (1995-1997), mean (SD)	70.37 (6.87)	72.28 (5.18)	.01
Time to debut, years, mean (SD)	0	8.01 (3.92)	
Education, n (%)			.40
Primary	1251 (60.8)	35 (67.3)	
Completed secondary	578 (28.1)	12 (23.0)	
Completed upper secondary	228 (11.1)	5 (9.6)	
High sensitivity C-reactive protein (hsCRP),(<3 mg/dl) mean (SD)	2.17 (2.03)	1.95 (1.90)	.25
<70.6	*n* = 1135	*n* = 20	
hsCRP, mean (SD)	2.13 (2.05)	2.77 (1.93)	.08
≥70.6	*n* = 922	*n* = 32	
hsCRP, mean (SD)	2.23 (2.01)	1.44 (1.71)	.00
Creatinine (53-115 μmol/L), mean (SD)	90.91 (16.25)	87.73 (12.74)	.16
Cholesterol (3.5-6.5 mmol/L), mean (SD)	6.54 (1.22)	6.63 (1.97)	.60
Triglycerides (0.6-1.8 mmol/L), mean (SD)	1.90 (1.02)	2.03 (1.25)	.38
Non-fasting blood glucose (4.0-5.9 mmol/L), mean (SD)	5.90 (1.86)	5.80 (1.22)	.68
Body Mass Index (kg/m^2^) mean (SD)	27.12 (4.15)	26.87 (4.02)	.67
Pulse (beats/min), mean (SD)	72.01 (13.21)	71.48 (12.73)	.78
Systolic BP (mmHg), mean (SD)	151.81 (22.91)	157.40 (22.31)	.08
Diastolic BP (mmHg), mean (SD)	85.03 (12.74)	85.85 (13.63)	.65
Diabetes Mellitus, n (%)	133 (6.5)	2 (3.8)	.45
Myocardial Infarction, n (%)	173 (8.4)	2 (3.8)	.24
Angina Pectoris, n (%)	258 (12.5)	5 (9.6)	.53
Stroke, n (%)	85 (4.1)	1 (1.9)	.43
Daily Smoker, ever, n (%)	1191 (57.9)	27 (52.0)	.16
Subjective health status			.27
Poor, n (%)	48 (2.3)	1 (1.9)	
Not so good, n (%)	757 (36.8)	18 (34.6)	
Good, n (%)	1129 (54.9)	33 (63.5)	
Very good, n (%)	123 (6.0)		

^a^*P*-values are derived from *t* tests for continuous variables and x^2^ tests for the binary variables

Multiple logistic regression analyses were performed for the total sample; and repeated separately for age groups 60- 70.5, and ages greater than or equal to 70.6 at the time of HUNT 2. The results for hsCRP in the total sample are shown in the upper part of Table 2 (1), in the sample aged 60-70.5 (2), and aged greater and equal to 70.6 in the lower portion (3). Results for total dementia, mixed AD/VaD, and VaD are presented in Table 3. There was no association between hsCRP and the risk of developing AD in the total sample, but there were significant age interactions in multivariate analyses. Additional analyses were performed with age dichotomized according to median age; and results of logistic regression analyses are shown in sections (2) and (3) of Table 2. In participants between 60 and 70.5, an adverse association was observed between hsCRP and AD. Conversely, in participants between 70.6 and 94, there was an inverse association between hsCRP and AD. Additional adjustment for all covariates did not change the finding.

**Table 2:** Multiple logistic regression analyses on the association of hsCRP and Alzheimer disease (AD)

	AD	NC^a^	Debut AD 0.4-7 years later		NC^a^	Debut AD 7-15 years later		NC^a^
(1) Age ≥60^b^	*n = 2150*	52	(4) Age ≥ 60	*n = 2150*	23	(7) Age ≥ 60	*n = 2150*	29
Model 1^c^	.77 (.47-1.26)		Model 1	.80 (.39-1.67)		Model 1	.84 (.41-1.71)	
Model 2^d^	.75 (.46-1.24)		Model 2	.82 (.39-1.73)		Model 2	.80 (.39-1.64)	
Model 3^e^	.78 (.47-1.32)		Model 3	.86 (.39-1.86)		Model 3	.83 (.39-1.75)	
Model 4^f^	.82 (.49-1.38)		Model 4	.93 (.42-2.04)		Model 4	.83 (.39-1.77)	
hsCRP*Age	.93 (.87-.99)		hsCRP*Age	.88 (.80-.97)		hsCRP*Age	.59 (.70-4.70)	
hsCRP*Sex	.90 (.31-2.64)		hsCRP*Sex	1.24 (.27-5.73)		hsCRP*Sex	2.40 (.37-15.71)	
(2) Age 60-70.5^b^	*n = 1176*	20	(5) Age 60-70.5	*n = 1176*	6	(8) Age 60-70.5	*n = 1176*	14
Model 1	1.85 (.89-3.85)		Model 1	4.20 (1.05-16.77)		Model 1	1.31 (.50-3.39)	
Model 2	1.83 (.85-3.93)		Model 2	5.77 (1.32-25.35)		Model 2	1.12 (.41-3.02)	
Model 3	2.34 (1.02-5.35)		Model 3	11.32 (2.01-63.67)		Model 3	1.29 (.44-3.80)	
Model 4	2.37 (1.01-5.58)		Model 4	14.20 (1.80- 112.22)		Model 4	1.29 (.42-3.96)	
hsCRP*Age	1.10 (.82-1.46)		hsCRP*Age	1.07 (.59-1.95)		hsCRP*Age	1.58 (1.03-2.42)	
hsCRP*Sex	2.40 (.37-15.71)		hsCRP*Sex	9.02 (.15-552.04)		hsCRP*Sex	1.67 (.48-5.78)	
(3) Age 70.6-94^b^	*n = 974*	32	(6) Age 70.6-94	*n = 974*	17	(9) Age 70.6-94	*n = 974*	15
Model 1	.36 (.18-.74)		Model 1	.35 (.13-.93)		Model 1	.48 (.16-1.46)	
Model 2	.36 (.18-.73)		Model 2	.33 (.13-.89)		Model 2	.50 (.16-1.5)	
Model 3	.35 (.17-.74)		Model 3	.31 (.11-.84)		Model 3	.53 (.17-1.68)	
Model 4	.39 (.19-.84)		Model 4	.34 (.12-.96)		Model 4	.54 (.16-1.81)	
hsCRP*Age	.93 (.77-1.13)		hsCRP*Age	.77 (.58-1.03)		hsCRP*Age	1.07 (.82-1.39)	
hsCRP*Sex	.65 (.14-2.99)		hsCRP*Sex	.64 (.08-4.97)		hsCRP*Sex	1.54 (.08-30.13)	

Results of the total sample (age ≥ 60) are shown in the upper left section (1), in age group 60-70.5 in middle left section (2) and in age group >70.6 in lower left section (3). Section 4-9 show analyses according to time from baseline (HUNT 2) to debut of AD, in different age groups

^a^Number of dementia cases

^b^Age when examined in HUNT 2

^c^Model 1: log transformed high specificity C-reactive protein (hsCRP)

^d^Model 2: hsCRP, age, sex, education

^e^Model 3: hsCRP, age, sex, education, cholesterol, triglycerides, non-fasting blood glucose, creatinine, body mass index, pulse

^f^Model 4: SBP, age, sex, education, cholesterol, triglycerides, non-fasting blood glucose, glomerular filtration rate, body mass index, pulse, history of myocardial infarction, diabetes mellitus, angina, stroke, smoking, subjective health status

**Table 3:** Complete analysis using logistic regression in examining the association of log transformed high sensitivity C reactive protein (hsCRP) and dementia

	Total Dementia	NC^a^	Combined AD, Mixed AD and Vascular Dementia	NC^a^	Alzheimer Disease	NC^a^	Mixed AD and Vascular Dementia	NC^a^
(1) Study sample *N* = 7758	HUNT 2	102	HUNT 2	84	HUNT 2	55	HUNT 2	29
Model 1^b^	1.55 (1.13-2.12)		1.57 (1.11-2.21)		1.23 (.79-1.90)		2.41 (1.37-4.25)	
Model 2^c^	1.02 (.72-1.44)		1.01 (.69-1.49)		.74 (.45-1.22)		1.71 (.92-3.19)	
Model 3^d^	1.03 (.71-1.48)		1.02 (.68-1.52)		.79 (.47-1.31)		1.60 (.84-3.06)	
Model 4^e^	1.01 (.70-1.45)		1.02 (.68-1.54)		.81 (.48-1.35)		1.55 (.80-2.97)	
hsCRP*Age	.98 (.95-1.00)		.98 (.95-1.01)		.98 (.94-1.02)		.98 (.93-1.03)	
hsCRP*Sex	.83 (.40-1.72)		.86 (.38-1.93)		.93 (.32-2.69)		.56 (.16-2.03)	
(2) <60^f^ *N* = 5608		9		7		3		4
Model 1	2.12 (.77-5.85)		1.83 (.57-5.90)		.08 (.00-3.71)		6.00 (1.29-28.00)	
Model 2	1.75 (.59-5.24)		1.40 (.39-5.04)		.06 (.00-2.96)		5.39 (.93-31.18)	
Model 3	2.43 (.79-7.47)		1.87 (.50-7.03)		.06 (.00-7.13)		5.26 (.82-33.72)	
Model 4	1.88 (.58-6.14)		1.53 (.37-6.25)		.05 (.00-8.31)		5.14 (.71-37.45)	
hsCRP*Age	1.06 (.90-1.26)		1.38 (1.01-1.90)		1.00 (.61-1.66)		.86 (.23-3.27)	
hsCRP*Sex	.24 (.02-2.69)		.96 (.56-1.63)				.06 (.00-10.03)	
(3) ≥60^f^ *N* = 2150		93		77		52		25
Model 1	.90 (.62-1.29)		.92 (.62-1.37)		.77 (.47-1.26)		1.33 (.68-2.59)	
Model 2	.89 (.62-1.28)		.91 (.61-1.36)		.75 (.46-1.24)		1.31 (.67-2.58)	
Model 3	.98 (.57-1.67)		.90 (.59-1.37)		.78 (.47-1.32)		1.19 (.46-3.09)	
Model 4	.88 (.60-1.29)		.92 (.61-1.41)		.82 (.49-1.38)		1.21 (.59-2.48)	
hsCRP*Age	.96 (.91-1.01)		.95 (.90-1.01)		.93 (.97-.99)		1.01 (.91-1.11)	
hsCRP*Sex	.99 (.46-2.12)		.92 (.39-2.14)		.90 (.31-2.64)		.77 (.19-3.17)	

^a^Number of dementia cases

^b^Model 1: log transformed high specificity C-reactive protein (hsCRP)

^c^Model 2: hsCRP, age, sex, education

^d^Model 3: hsCRP, age, sex, education, cholesterol, triglycerides, non-fasting blood glucose, creatinine, body mass index, pulse

^e^Model 4: SBP, age, sex, education, cholesterol, triglycerides, non-fasting blood glucose, glomerular filtration rate, body mass index, pulse, history of myocardial infarction, diabetes mellitus, angina, stroke, smoking, subjective health status

^f^Age when examined in HUNT 2

Additional analyses were performed and presented in Table 2 (4-9) examining whether the number of years to AD onset from baseline influenced the association between hsCRP and AD. A similar adverse trend was observed amongst the sample diagnosed 0.4 to 7 years following hsCRP measurement. Amongst those 60-70.5, the adverse association between hsCRP and AD was attenuated and did not retain significance. An opposite trend was observed amongst those ≥70.6, where an inverse association was observed in participants diagnosed up to seven years later. The inverse association between hsCRP and AD amongst those diagnosed with AD seven-15 years later was attenuated and did not retain significance.

## Discussion

The main finding of our study was that hsCRP levels were adversely associated with participants aged between 60 and 70.5, and inversely associated with developing AD in participants aged ≥70.6. When applying multivariate models the findings were significant in individuals diagnosed only 0.4-7 years after the hsCRP was measured; and attenuated when AD was diagnosed more than seven years following hsCRP measurement.

Our findings support previous studies that report contrast findings when considering age. As in previous studies, participants in the younger age bracket (60-70.5) advocated that high hsCRP was associated with an increased risk of AD [13]. In oldest participants, our findings support previous studies reporting an inverse association between hsCRP and AD [14, 15, 18, 19, 26–30].

Our study had a number of strengths in comparison with earlier studies, as a large number of subjects over the age of 60 had a follow-up time of up to 16 years. In addition, the prospective study design allowed for extensive control for numerous chronic conditions. Also, the utilized hsCRP assay has been shown to be a peripheral biomarker with high assertion. Our study should, however, be interpreted with some limitations. The HUNT Study participants are mostly Caucasian and the population is well educated, and results may not apply to all ethnicities or social demographics. The sample sizes in stratified analyses were relatively small. Although efforts were made to identify participants diagnosed with dementia in the region during 1995–2011 by performing hospital record searches and examining nursing home residents, we had no access to data from individuals with dementia who were under the care of their general practitioner, and these will appear as false-negatives in the data set. However, the proportion of false-negatives to true-negatives in the non-case group is quite low because the prevalence of dementia is, after all, low. Therefore, the contamination of the non-case group will not be substantial, and the effect estimates will be little more than inconsequential. Lastly, the prescription registry was not linked with the current study, and we cannot exclude that medication had an influence on hsCRP values, as it has been known that NSAIDs and lipid lowering medication such as statins reduce hsCRP values [31].

One challenge of the present study is to understand why hsCRP are in contrast when examining age of the participant during the years hsCRP is observed until the AD onset. It is questionable whether lower hsCRP values provides protection from AD, or if it is the result of the neuropathology in older at-risk individuals. A recent meta-analysis of CRP in persons with AD discussed whether CRP levels could be different in different stages of the disease trajectory. The authors speculated whether CRP is decreased in mild or moderate AD, and increased in the following severe stage [32]. Dementia disorders are progressive and fatal disorders, as the blood samples obtained were an average of 8 years prior to diagnosis, it must be assumed that these were taken before AD developed or in very early stages. The results in this study appear to be more dependent on the age of the participant.

There have been a number of studies examining how immune responses can be affected by the pathophysiology of AD. Advances in neuroimmunology have shown that the molecular innate immune response is dysfunctional in AD [33]. The body’s immune response in AD responds to an aggregation of amyloid-β (Aβ) peptides in the endoplasmic reticulum (ER) that causes stress and activation of the unfolded protein response (UPR) [34]. UPR aims to alleviate stress and minor elevations of systemic inflammatory markers, reflects the presence of stressed cells. In circumstances of chronic or prolonged ER stress, sensors responsible for binding to misfolded proteins change from acting pro-protective to pro-apoptotic [35]. It has been postulated that the molecular mechanisms involved in the innate immune response are disrupting UPR functioning and can be involved in the pathogenesis of AD [34]. Although the precise molecular pathways of neuroinflammation remain unclear, a gene expression study found inflammatory changes in the aging brain regarded as age-dependent [17]. Interestingly, the period between the sixth and seventh decade was observed to undergo robust gene expression changes.

It is known that clinical AD is preceded by decades of a prodromal phase. During this asymptomatic phase, systemic changes are known to be occurring. To examine whether our findings were influenced by ascertainment time, samples were split by the number of years participants developed AD following hsCRP measurement, see Table 2 (sections 4-9). There was a stronger association in participants who were diagnosed up to seven years later in comparison with those who were diagnosed seven to 15 years later. However, sample sizes in these stratified analyses were small and it is questionable whether the finding is a true association or the result of preclinical AD. Although, participants with dementia were ascertained in both nursing home and residential settings, it is perhaps speculative to say that nursing home participants were in a more severe stage than those at home, as there can be many other factors determining whether a Norwegian resident needs placement in nursing care. For example, those living in secluded areas, and often alone are demanding admission to a nursing home facility sooner than residents living at home with help from family and regardless of the stage severity. It is therefore difficult to distinguish strictly on this basis. Therefore, we examined stage severity using years to onset. Since the hsCRP marker was taken an approximately 8 years prior to diagnosis, it is most likely these participants were not exhibiting cognitive decline or at most, mild cognitive impairment.

Finally, low-grade inflammation is defined as being a state where the body is constantly under very mild chronic inflammation but not to the extent of acute inflammation. Minor elevation in inflammatory markers are measured in blood with inflammatory markers, such as hsCRP. Defining a precise cut-off between these two states is difficult, but many previous studies define a hsCRP under 10 with low-grade inflammation; and values above this as clinically significant inflammatory states [5]. The American Heart Association have suggested that cut points of hsCRP below 1 mg/l, between 1 and 3 mg/l, and greater than 3 mg/l can be used to find those at lower, average, and high relative risk for CVD events [36]. Replication of our data will strengthen the existing evidence whether similar cut points of hsCRP, in addition to a panel of other inflammatory markers, such as interleukins, should be considered clinically relevant when monitoring patients at risk for dementia.

## Conclusions

Our study is in line with previous studies indicating a shift in the association between hsCRP and AD by age: in adults (60-70.5 years) there is an adverse association, while in seniors (>70.6 years) there is an inverse association. Regardless that the nature of the association remains unclear, our data and data from preclinical and clinical studies have established the immune system-mediated actions contribute and drive AD pathogenesis [1]. Continued research in persons at risk is needed to advance the role inflammation has in AD. If our findings can be replicated, future intervention studies should assess whether medical treatment of low-grade inflammation will reduce incidence of AD. More studies are needed to further examine why a more active peripheral immune response may have a protective role in individuals ≥70 years.

## Abbreviations

AD: Alzheimer disease
Aβ: Amyloid beta
BMI: Body mass index
CI: Confidence interval
CRP: C reactive protein
CV: Coefficient of variation
DM: Diabetes mellitus
ER: Endoplasmic reticulum
HMS: Health and Memory Study of Nord-Trøndelag (1995–2010)
hsCRP: High specificity C reactive protein
HUNT 1: Helse Undersøkelse Nord-Trøndelag (1984–1986)
HUNT 2: Helse Undersøkelse Nord-Trøndelag (1995–1997)
HUNT 3: Helse Undersøkelse Nord-Trøndelag (2006-2008)
ICD-10: International Classification of Diseases, Tenth Revision
MI: Myocardial infarction
MW: Mann Whitney
NSAIDs: Non steroidal anti inflammatory drugs
OR: Odds ratio
T2D: Type two diabetes mellitus
UPR: Unfolded protein response
VaD: Vascular dementia
